# Identification of southern Taiwan genetic variants in thyroid dyshormonogenesis through whole‐exome sequencing

**DOI:** 10.1002/kjm2.12871

**Published:** 2024-06-25

**Authors:** Ching‐Chao Tsai, Yu‐Ming Chang, Yen‐Yin Chou, Shou‐Yen Chen, Yu‐Wen Pan, Meng‐Che Tsai

**Affiliations:** ^1^ Department of Pediatrics, National Cheng Kung University Hospital, College of Medicine National Cheng Kung University Tainan Taiwan; ^2^ Department of Genomic Medicine, National Cheng Kung University Hospital, College of Medicine National Cheng Kung University Tainan Taiwan; ^3^ Department of Pediatrics Kuo General Hospital Tainan Taiwan; ^4^ Department of Medical Humanities and Social Medicine, School of Medicine, College of Medicine National Cheng Kung University Tainan Taiwan

**Keywords:** congenital hypothyroidism, genetic variation, thyroid diseases, thyroid dyshormonogenesis, whole exome sequencing

## Abstract

Thyroid dyshormonogenesis (TDH) is responsible for 15%–25% of congenital hypothyroidism (CH) cases. Pathogenetic variants of this common inherited endocrine disorders vary geographically. Unraveling the genetic underpinnings of TDH is essential for genetic counseling and precise therapeutic strategies. This study aims to identify genetic variants associated with TDH in Southern Taiwan using whole exome sequencing (WES). We included CH patients diagnosed through newborn screening at a tertiary medical center from 2011 to 2022. Permanent TDH was determined based on imaging evidence of bilateral thyroid structure and the requirement for continuous medication beyond 3 years of age. Genomic DNA extracted from blood was used for exome library construction, and pathogenic variants were detected using an in‐house algorithm. Of the 876 CH patients reviewed, 121 were classified as permanent, with 47 (40%) confirmed as TDH. WES was conducted for 45 patients, and causative variants were identified in 32 patients (71.1%), including DUOX2 (15 cases), TG (8 cases), TSHR (7 cases), TPO (5 cases), and DUOXA2 (1 case). Recurrent variants included DUOX2 c.3329G>A, TSHR c.1349G>A, TG c.1348delT, and TPO c.2268dupT. We identified four novel variants based on genotype, including TSHR c.1135C>T, TSHR c.1349G>C, TG c.2461delA, and TG c.2459T>A. This study underscores the efficacy of WES in providing definitive molecular diagnoses for TDH. Molecular diagnoses are instrumental in genetic counseling, formulating treatment, and developing management strategies. Future research integrating larger population cohorts is vital to further elucidate the genetic landscape of TDH.

## 1 INTRODUCTION

Congenital hypothyroidism (CH) is a disorder marked by a deficiency in thyroid hormones from birth. If left untreated, hypothyroidism can lead to severe intellectual disabilities. CH ranks among the most prevalent endocrine disorders in newborns,^1^ with an incidence of one in 2500–3000 births.^2^ The etiology of CH has been traditionally classified as thyroid dysgenesis (TD) or thyroid dyshormonogenesis (TDH). The majority of TD cases are sporadic, while most TDH cases are autosomal‐recessively inherited from parents carrying a heterozygous pathogenic allele. Recent advancements in genomic medicine have led to the discovery of factors and genes responsible for thyroid hormone production and associated abnormalities. Most TDH cases are attributed to mutations in *SLC26A4*, *DUOX1*, *DUOX2*, *DUOXA2*, *TPO*, *TG*, *TSHR*, *IYD*, and *SLC26A7*, which are integral to thyroid hormone production and predominantly inherited in an autosomal recessive manner.^3^

Traditionally, the etiology of CH has been attributed to TD in 75%–85% of cases, while TDH accounts for 15%–25%.^4^ However, the results of several studies conducted worldwide to evaluate the incidence of CH and TDH in the recent decade vary considerably.^5^ Some studies report an increasing trend in CH or TDH, as in India and Japan,^5^, ^6^ while others have shown no such trend, as in Finland.^7^ Several factors have been proposed to explain the observed upward trend in the incidence of CH or TDH. These factors include the lowering of the thyrotropin cut‐off value for CH, the increased survival of extremely preterm infants, who are at greater risk for CH, and the higher prevalence of consanguineous families in several countries. Demographic shift is also found to contribute to the increased incidence of CH, although the reasons for this trend remain unclear in certain studies. Furthermore, demographic shifts may contribute to the emergence of distinct genetic hotspots among CH patients, particularly those with TDH, in specific regions. For example, the variant c.1588A>T in the *DUOX2* gene was found to be widespread in the Thai population.^8^ In Taiwan, several recurrent mutations have been reported.^9^, ^10^, ^11^ These include *TSHR* p.R450H, a missense variant found in approximately 7% of CH patients in Southern Taiwan.^9^ Additionally, *TG* c.1348delT and *TPO* 2268insT have been identified as common mutations resulting from founder effects.^10^, ^11^ However, it is worth noting that these studies conducted genetic testing using a single‐gene approach as opposed to high‐throughput sequencing, which is widely applied in the study of diseases caused by multiple genes. Therefore, our objective is to conduct a comprehensive review of CH cases and evaluate the genetic etiology of TDH patients in a single tertiary center in Southern Taiwan. To the best of our knowledge, this study represents the first whole‐exome sequencing (WES) analysis comprehensively examining the spectrum of mutations in patients with TDH in Taiwan. Considering the needs for genetic counseling and guidance for choosing medication strategies, a molecular diagnosis may help to answer the concerns of parents of CH patients. Due to the genetic heterogeneity of CH, WES has now become widely used to provide high‐throughput sequencing data for CH, thereby facilitating genetic diagnosis for clinical use. This study aims to identify the etiological genetic variants of TDH in our patient cohort in order to elucidate the local genetic profile of TDH in Southern Taiwan.

## 2 METHODS

### 2.1 Cohort and sample collection

**FIGURE 1:**
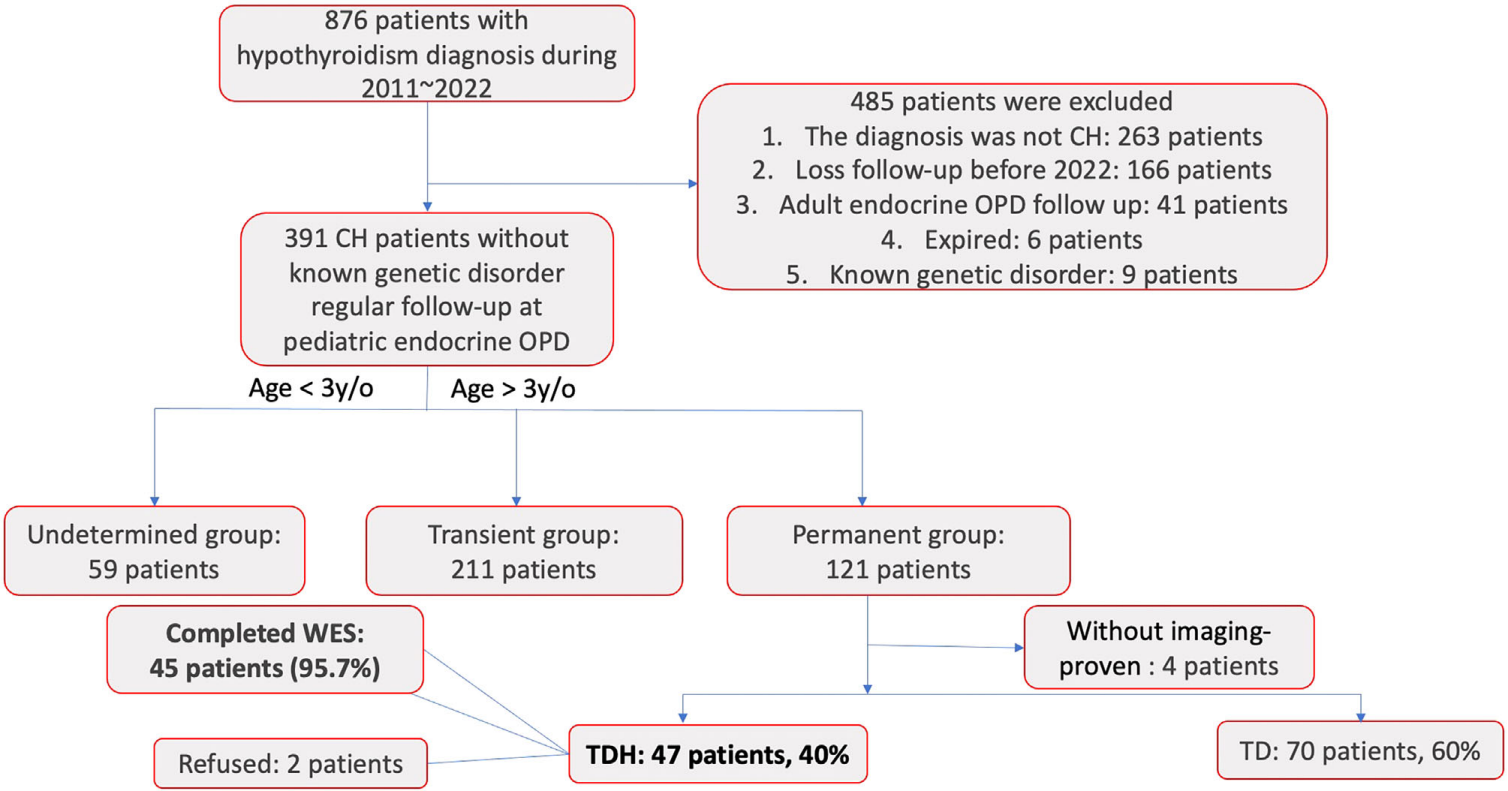
A total of 45 patients was selected from the flowchart.

We screened patients diagnosed with and treated for hypothyroidism at our medical center from 2011 to 2022. CH was diagnosed based on neonatal screening results indicating an initial thyroid stimulating hormone (TSH) level exceeding 10 μU/mL. Excluded from the study were patients who were not diagnosed with CH, lost to follow‐up before 2022, transferred to adult endocrinology care, deceased, or diagnosed with other known genetic disorders. The eligibility criteria for permanent TDH diagnosis included the imaging‐confirmed presence of bilateral normal thyroid structures and the requirement for thyroxine supplementation for a minimum of 3 years after birth. Tc99m was generally the preferred imaging modality; however, if the patient's family had concerns regarding radiation exposure, a thyroid ultrasound was used as an alternative. All TDH patients who were identified through the screening process were invited to participate in the study. The final cohort included 45 patients born to non‐consanguineous parents. Written informed consent was provided by patients or their legal guardians, as appropriate (refer to Figure 1). The study was approved by the Ethics Committee (IRB number: B‐BR‐111‐012).

### 2.2 Whole‐exome sequencing

The exome library was constructed using genomic DNA extracted from the patient's peripheral blood. An in‐house algorithm was employed for identifying pathogenic variants. The samples were sequenced using the Illumina DNA Prep with Enrichment, followed by the NextSeq 2000, generating 2 × 150 bp paired‐end reads. Sequencing was conducted at our hospital's facility. In our protocol, matching cases for combined bioinformatic analysis was crucial, as was the use of one specific version of the exon capture kit. Different kits vary in coverage, affecting well‐documented and newly annotated genes, including non‐protein‐coding genes such as lncRNAs.

### 2.3 Congenital‐hypothyroidism–related gene analysis

For genetic sequence analysis, variants associated with TDH were extracted from our refined exome dataset, as detailed in Table 1. Initially, a quality control depth greater than 20 was established, along with a minor allele frequency threshold of 0.01, using data from the Genome Aggregation Database, the 1000 Genomes Project, and the Taiwan Biobank. The ClinVar and Leiden Open Variation Databases were consulted for known pathogenic variants. Predictive algorithmic tools including PolyPhen‐2 HVAR (http://genetics.bwh.harvard.edu/pph2/), SIFT (https://sift.bii.a-star.edu.sg/), VEST3 (https://jhu.technologypublisher.com/technology/24805), Mutation Taster (https://www.mutationtaster.org/), MetaSVM, MetaLR, DANN (https://cbcl.ics.uci.edu/public_data/DANN/), dbscSNV_ada (http://www.liulab.science/dbscsnv.html), dbscSNV_rf (http://www.liulab.science/dbscsnv.html), and spidex (https://www.openbioinformatics.org/annovar/spidex_download_form.php) were employed to identify potentially pathogenic variants. Combined Annotation Dependent Depletion (CADD) (https://cadd.gs.washington.edu/) scores were also used, setting a threshold value ≥20 for evaluating patients. The guidelines of the American College of Medical Genetics and Genomics (ACMG)^12^ were applied in some cases. Only pathogenic variants and those of uncertain significance were retained for this analysis.

**TABLE 1:** CH‐related genes.

Gene name	Gene ID	Gene name	Gene ID	Gene name	Gene ID	Gene name	Gene ID
NKX2‐1	7080	HOXA3	15,400	GATA6	2627	TG	7038
TSHR	7253	HES1	15,205	KDM6A	7403	TRPC4AP	26,133
TPO	7173	EYA1	2138	MC2R	4158	PAX8	7849
DUOX1	53,905	TUBB1	81,027	MRAP	56,246	HHEX	3087
KMT2D	8085	GLIS3	169,792	NNT	23,530	FOXE1	2304
SLC5A5	6528	JAG1	182	PDE4FD	5144	FGF8	14,179
DUOXA2	405,753	CDCA8	55,143	PLAA	9373	TBX1	21,380
DUOX2	50,506	NKX2‐5	1482	PRKAR1A	5573	BCL2L1	598
IYD	389,434	NTN1	18,208	STAR	6770	ATP6V1B2	526
SLC26A4	5172	THRB	7068	TBC1D24	57,465	B3GLCT	145,173
ANO1	55,107	HAND2	15,111	THRA	21,833	TSHB	7252
SLC26A2	6567	FGF1	2246	TONSL	4796	TXNRD2	10,587
DUOXA1	90,527	FGF2	2247	TRAPPC9	83,696	YRDC	79,693
ZBTB20	56,490						

**TABLE 2:** Comparison of patient characteristics between positive and negative gene variant groups.

	Positive gene variant group (mean value, standard deviation)	Negative gene variant group (mean value, standard deviation)	*p*
Sex			0.341
Male	20 (62.5%)	6 (46.2%)	
Female	12 (37.5%)	7 (53.8%)	
Age at diagnosis of CH (years)	22.1, 14.9	32.3, 21.8	0.503
Initial thyroxine dosage (μg/kg/day)	13.9, 2.7	13.8, 2.5	0.447
Mean TSH (mIU/L)	118.3, 124.5	123.4, 144.7	0.457
Mean T4 (μg/dL)	5.8, 2.8	5.6, 3.1	0.431
Mean T3 (ng/dL)	122.3, 43.2	126.9, 38.8	0.392
BBW (grams)	2985.6, 432.1	2541.2, 884.7	0.020

### 2.4 Statistical analysis

Student's *t*‐test was used to compare initial thyroid function, birth body weight, age at diagnosis of CH, and initial thyroxine dosage between groups with and without gene mutations. Grubbs' test was performed to determine whether the most extreme value was a significant outlier. Fisher's exact test compared sex probabilities between the positive and negative gene variant groups. Statistical significance was set as *p* < 0.05.

## 3 RESULTS

Of the 876 CH patients reviewed, 121 were classified as having permanent CH; TDH was confirmed in 47 (40%) of the permanent CH cases. Four cases were undetermined due to the absence of imaging. Of the 47 TDH patients, 45 (95.7%) consented to participate in the study, including three pairs of siblings (patients 5 and 6, 8 and 9, and 27 and 28). Of these 45 patients, 26 (57.8%) were male and 19 were female (42.2%). 34 were born full‐term, and 6 were preterm infants. The remaining 5 cases could not recall their gestational age. WES identified causative variants in 32 patients (71.1%). No significant differences were found between patients with and without identified genetic etiologies in terms of sex, gestational age, age at CH diagnosis, initial thyroxine dosage, TSH, thyroxine (T4) level, or triiodothyronine (T3) level. Notably, birth body weight differed significantly between the two groups (*p* = 0.020), and this difference persisted even after using Grubbs' test to determine if the most extreme value was a significant outlier (Table 2). We identified four novel variants through WES: *TSHR* c.1135C>T, *TSHR* c.1349G>C, *TG* c.2461delA, and *TG* c.2459T>A.

**FIGURE 2:**
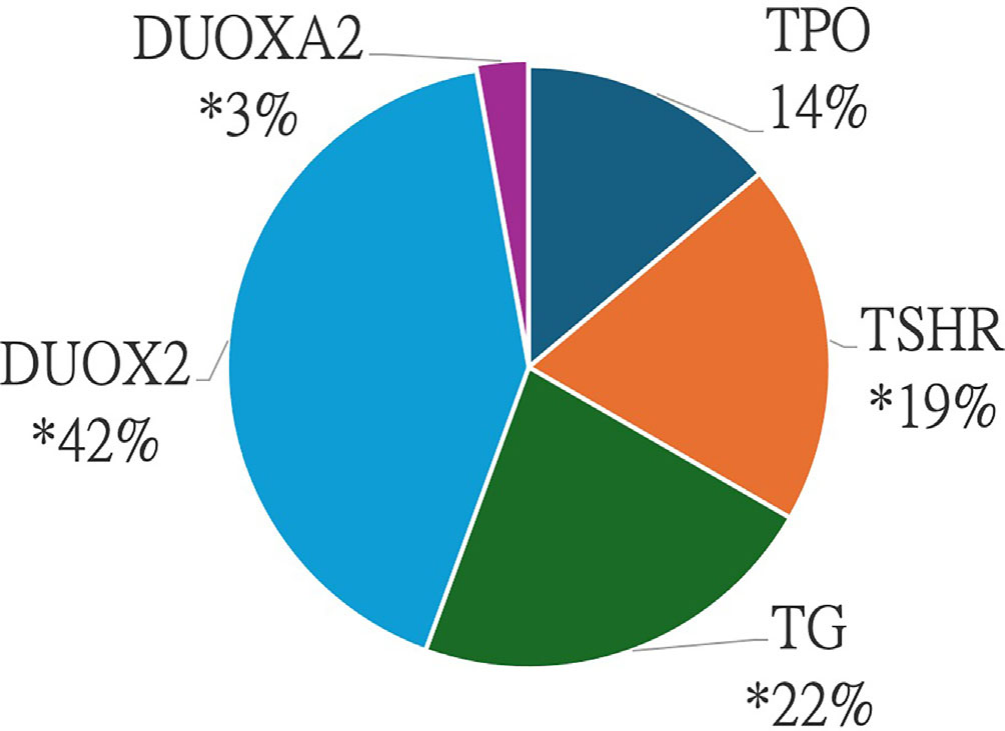
Percentage of causative variants of each gene relative to the total causative variants. *32 patients total, of which four exhibited digenic gene mutations, resulting in a collective count of 36 mutations.

The variants found in this study were located in *DUOX2* (15 cases), *TG* (8 cases), *TSHR* (7 cases), *TPO* (5 cases), and *DUOXA2* (1 case) (Figure 2). Of these cases, four exhibited digenic mutations. The characteristics of the TDH patients are shown in Table 3. The most common mutations in our TDH patient cohort were *DUOX2* c.3329G>A (p.R1110Q) (13.3%), *TSHR* c.1349G>A (p.R450H) (11.1%), *TPO* c.2268dupT (p.E757*) (8.9%) and *TG* c.1348delT (p.S450) (8.9%), suggesting recurrent hotspots among our TDH patients (Table 4).

**TABLE 3:** Clinical data for TDH patients.

Patient ID	Age/sex	Age at diagnosis	TSH (mIU/L)	T4 (μg/dL)	T3 (ng/dL)	GA (week)	Echo/Technetium‐99 m	Associated features	BBW (g)	BL (cm)	BW (kg)	BMI (kg/m^2^)	Initial dose (μg/kg)
1	10/M	D15	38.1	9.8	140.6	38	Tc99m	Precocious puberty	3000	145.2	43.8	20.8	12.5
2	26/M	D37	127.4	3.7	34.2	X	Echo	‐	2500	161.5	51.7	19.8	10
3	21/F	3 m	25.1	6.8	135.6	39	Echo	‐	2750	151.8	43.1	18.7	10.2
4	22/F	D26	240	0.1	41.3	Term	Echo	‐	3100	161.5	47.8	18.3	14.7
5	8/M	D15	73.7	9.8	151.9	Term	Tc99m	Overweight	3100	131.1	33.4	19.4	14.7
6	7/F	D18	91	7.2	157.1	Term	Tc99m	‐	2960	124.8	21.8	14	14.7
7	15/F	3 m	52.1	10.6	116	38	Tc99m	‐	2752	160	49.6	19.4	9.8
8	10/M	D24	72.6	6.2	115.5	X	Tc99m	‐	2510	145.9	31.7	14.9	16.7
9	10/M	D35	59.8	7.6	150.5	X	Tc99m	‐	2710	144.7	31.2	14.9	13.2
11	17/M	D21	51.2	6.7	121.4	40	Tc99m	1. Overweight	3400	162.6	61.6	23.3	11.1
								2. Precocious puberty					
12	26/F	D34	187.5	4.9	139.1	Term	Tc99m	‐	3200	172	56.4	19.1	12.2
13	33/M	X	82.8	6.2	166	Term	Echo	Overweight	X	180.5	92.7	28.5	X
14	7/M	D17	31.2	8.5	144.6	Term	Echo	Toureet syndrome	3020	132.6	27.9	15.9	13.9
15	4/F	X	X	X	X	38+6	Echo	‐	2850	110.6	17.2	14.1	X
16	5/F	D27	137.7	3.1	95.2	36+1	Tc99m	‐	2500	116.5	21.1	15.5	14.3
17	5/F	X	X	X	X	37	Tc99m	Poor body weight gain	2300	109.1	15.1	12.7	X
18	7/M	D10	448.8	0.8	56.4	39	Tc99m	‐	3600	120	23	16	13.9
19	13/F	D27	233.5	1.9	140.2	40	Tc99m	1. Precocious puberty	3154	156.5	54.3	22.2	11.9
								2. Right goiter					
20	4/M	D16	39.6	9.7	145.7	Term	Tc99m	1. Autism	3389	115	17.1	12.9	14.7
								2. DevelopmentDelay					
21	5/M	D26	42.8	7.3	135.5	Term	Tc99m	Short stature	3440	117.1	18.9	13.8	11.4
22	4/M	D15	50.3	5.8	147.6	39+6	Tc99m	‐	3492	111.8	18.8	15	13.9
23	4/M	D25	47.1	4.1	187.2	Term	Echo	Atopic dermatitis	3030	110.1	18.5	15.3	12.5
25	10/M	D28	10.2	10.8	130.1	37	Tc99m	‐	3420	149.2	53.7	24.1	12.8
26	31/F	D7	443	0.8	X	41	Tc99m	‐	3600	159	42.9	17	13.5
27	3/F	D7	484.6	5.5	132.7	36+6	Tc99m	‐	2570	104	15.6	14.4	19.2
28	3/M	D7	14.9	2.1	25.7	36+6	Echo	1. Autism	1790	95.7	11.4	12.4	13.2
								2. Short stature					
								3. Left thyroid nodule					
30	3/F	D26	23.3	8.4	168.5	Term	Echo	1. Premature thelarche	3425	99.9	15.6	15.6	11.9
								2. Poor body weight gain					
31	26/M	D26	23.8	7.5	167.1	X	Tc99m	‐	X	172	60.1	20.3	X
32	6/F	D62	10.5	2.9	X	24+3	Echo	1. Short stature	669	106	13.1	11.7	13.9
								2. Short bowel syndrome					
33	8/M	D11	442.4	3.2	112.7	40	Tc99m	‐	3330	130	29.1	17.2	16.7
35	7/M	D27	79.6	5.2	232.1	40	Tc99m	Poor body weight gain	2904	127.5	22.2	13.7	13.5
36	6/M	D13	326.5	<0.8	31.6	27+3	Echo	Language delay	1220	114.8	18.5	14	15
37	4/M	D14	63.4	7.3	132.3	38	Tc99m	‐	3310	105.6	15.8	14.2	13.9
38	3/F	D26	187.4	2.9	91.5	X	Tc99m	‐	X	96.9	14.9	15.9	11.4
39	13/M	D30	X	X	X	33+1	Tc99m	1. Epilepsy	1400	121	25.9	17.7	X
								2. Cerebral palsy					
								3. Estropia					
40	6/M	D20	170.4	4.4	122.7	37	Tc99m	Short stature	3200	114.1	21.7	16.6	11.9
41	4/M	D15	42.3	9.2	155.1	39	Tc99m	1. Autism	3075	99.1	14.9	15.2	14.3
								2. Development delay					
42	30/M	D17	21.3	1.2	X	Term	Echo	Obesity	3675	166.5	89.3	32.2	9
43	9/M	D20	120	2.1	106.2	38	Tc99m	Short stature	3050	134.3	36.1	20	11.4
44	31/F	D31	>100	4	121	Term	Echo	Iron deficiency anemia	3300	164	78.5	29.2	13.2
45	6/F	D8	77.4	9	105.1	38	Tc99m	‐	2576	114.7	20	15.2	20
46	3/F	D7	106.2	7.3	132.4	Term	Tc99m	‐	2703	93.8	14.6	16.6	18.5
47	6/M	D23	22.2	6.2	111.7	39+1	Echo	1. Atopic dermatitis	2002	116.5	21.7	16	18.5
								2. Developmentdelay					
								3. Epilepsy					
49	10/F	D33	13.7	7.4	125.6	38+3	Tc99m	Precocious puberty	2740	148	38.3	17.5	18.2
50	16/F	D25	89.4	5.6	85.8	40	Tc99m	Endocardial cushion defect	2900	160	49.1	19.2	17.2

Abbreviations: BBW, birth body weight; BL, body length; BMI, body mass index; BW, body weight; F, female; GA, gestational age; M, male; T3, triiodothyronine; T4, thyroxine; X, no data available; −, negative findings.

**TABLE 4:** Molecular and protein descriptions of variants found in TDH patients.

Patient ID	Gene	Variant position	Amino acid change	Exon	Homozygous/heterozygous	Variant type	Variant name‐MAF/gnomAD	Clinvar	ACMG	Mode of inheritance
2	TPO	c.2268dupT	p.E757*	Exon13	Homozygous	Nonsense	gnomAD: 0.0001	Pathogenic	Pathogenic	AR
							TW: 0.003504			
4	TPO	c.2268dupT	p.E757*	Exon13	Homozygous	Nonsense	gnomAD: 0.0001	Pathogenic	Pathogenic	AR
							TW: 0.003504			
5	TSHR	c.1349G>A	p.R450H	Exon10	Heterozygous	Missense	gnomAD: 0.000235	Conflicting	VUS	AR
							1000G: 0.0002			
							TW: 0.002503			
6	TSHR	c.1349G>A	p.R450H	Exon10	Heterozygous	Missense	gnomAD: 0.000235	Conflicting	VUS	AR
							1000G: 0.0002			
							TW: 0.002503			
7	TSHR	c.1349G>C	p.R450P	Exon10	Heterozygous	Missense	Not found	1. ‐	1. Pathogenic	AR
	TSHR	c.1349G>A	p.R450H	Exon10	Heterozygous	Missense	gnomAD: 0.000235	2. Conflicting	2. VUS	AR
							1000G: 0.0002			
							TW: 0.002503			
8	DUOX2	c.2654G>A	p.R885Q	Exon20	Heterozygous	Missense	gnomAD: 0.000115	1. Pathogenic	1. Pathogenic	AD AR
	DUOX2	c.2291G>A	p.R764Q	Exon18	Heterozygous	Missense	TW: 0.0005	2. VUS	2. VUS	AD AR
	TSHR	c.733G>A	p.G245S	Exon9	Heterozygous	Missense	1000G: 0.0002	3. Conflicting	3. VUS	AR
							gnomAD: 0.00000795			
							1000G: 0.0004			
							TW: 0.002			
9	DUOX2	c.2654G>A	p.R885Q	Exon20	Heterozygous	Missense	gnomAD: 0.000115	1. Pathogenic	1. Pathogenic	AD AR
	DUOX2	c.2291G>A	p.R764Q	Exon18	Heterozygous	Missense	TW: 0.0005	2. VUS	2. VUS	AD AR
	TSHR	c.733G>A	p.G245S	Exon9	Heterozygous	Missense	1000G: 0.0002	3. Conflicting	3. VUS	AR
							gnomAD: 0.00000795			
							1000G: 0.0004			
							TW: 0.002			
11	DUOX2	c.3329G>A	p.R1110Q	Exon25	Heterozygous	Missense	gnomAD: 0.0002	1. Conflicting	1. VUS	AD AR
	DUOX2	c.3391G>T	p.A1131S	Exon25	Heterozygous	Missense	1000G: 0.0004	2. ‐	2. VUS	AD AR
	DUOX2	c.2202G>A	p.W734*	Exon18	Heterozygous	Missense	TW: 0.004004	3. ‐	3. VUS	AD AR
							TW: 0.000501			
							TW: 0.000501			
13	TG	c.1351C>T	p.R451*	Exon9	Heterozygous Heterozygous	Missense	gnomAD: 0.0000159	1. ‐	1. VUS	AR
	TG	c.1348delT	p.S450	Exon9		FS	gnomAD: 0.0000558	2. ‐	2. Pathogenic	AR
							TW: 0.001502			
14	TPO	c.670_672del	p.D224del	Exon7	Heterozygous	Inframe deletion	TW: 0.0005	‐	1. VUS	AR
16	DUOX2	c.2654G>A	p.R885Q	Exon20	Heterozygous	Missense	gnomAD: 0.000115	1. Pathogenic	1. Pathogenic	AD AR
	DUOX2	c.3329G>A	p.R1110Q	Exon25	Heterozygous	Missense	TW: 0.000501	2. Conflicting	2. VUS	AD AR
							gnomAD: 0.0002			
							1000G: 0.0004			
							TW: 0.004004			
17	DUOX2	c.3329G>A	p.R1110Q	Exon25	Heterozygous	Missense	gnomAD: 0.0002	1. Conflicting	1. VUS	AD AR
	DUOX2	c.2101C>T	p.R701*	Exon17	Heterozygous	Missense	1000G: 0.0004	2. Pathogenic	2. Pathogenic	AD AR
							TW: 0.004004			
							gnomAD: 0.0000318			
							TW: 0.000501			
18	DUOX2	c.1588A>T	p.K530*	Exon14	Heterozygous	Missense	gnomAD: 0.0004	1. Pathogenic	1.Pathogenic	AD AR
	DUOX2	c.4561G>T	p.G1521*	Exon34	Heterozygous	Missense	1000G: 0.0006	2. VUS	2. VUS	AD AR
							TW: 0.013514			
							gnomAD: 0.0000719			
							TW: 0.001001			
20	TSHR	c.1154A>G	p.Y385C	Exon10	Heterozygous	Missense	gnomAD: 0.00000795	1. ‐	1. VUS	AR
	TSHR	c.1349G>A	p.R450H	Exon10	Heterozygous	Missense	gnomAD: 0.000235	2. Conflicting	2. VUS	AR
							1000G: 0.0002			
							TW: 0.002503			
21	DUOX2	c.2635G>A	p.E879K	Exon20	Heterozygous	Missense	gnomAD: 0.0000756	1. Pathogenic	1. Pathogenic	AD AR
	DUOX2	c.1772T>C	p.F591S	Exon15	Heterozygous	Missense	TW: 0.001	2. VUS	2. VUS	AD AR
							TW: 0.0005			
22	TG	c.5261C>G	p.S1754*	Exon27	Heterozygous	Missense	gnomAD: 0.0000199	1. ‐	1. VUS	AR
	TG	c.8085G>C	p.E2695D	Exon47	Heterozygous	Missense	gnomAD: 0.0000278	2. ‐	2. VUS	AR
27	TG	c.1351C>T	p.R451*	Exon9	Heterozygous	Missense	gnomAD: 0.0000159	1. ‐	1. VUS	AR
	DUOX2	c.3329G>A	p.R1110Q	Exon25	Heterozygous	Missense	gnomAD: 0.0002	2. Conflicting	2. VUS	AD AR
	DUOX2	c.1268C>T	p.T423I	Exon12	Heterozygous	Missense	1000G: 0.0004	3. Conflicting	3. VUS	AD AR
							TW: 0.0025			
							gnomAD: 0.0002			
							1000G: 0.0006			
							TW: 0.0005			
28	DUOX2	c.3329G>A	p.R1110Q	Exon25	Heterozygous	Missense	gnomAD: 0.0002	1. Conflicting	VUS	AD AR
							1000G: 0.0004			
							TW: 0.0025			
30	TG	c.2461delA	p.I821	Exon10	Heterozygous	Nonsense	Not found	1. ‐	1. VUS	AR
	TG	c.2459T>A	p.F820Y	Exon10	Heterozygous	Missense	Not found	2. ‐	2. VUS	AR
31	TG	c.274+2T>G	‐	Exon3	Heterozygous Heterozygous	Splicing	TW: 0.0005	1. ‐	1. VUS	AR
	TG	c.7364G>A	p.R2455H	Exon42	Heterozygous	Missense	gnomAD: 0.0005	2. Conflicting	2. VUS	AR
	TG	c.7753C>T	p.R2585W	Exon44		Missense	1000G: 0.0012	3. Conflicting	3. VUS	AR
	DUOXA2	c.93T>G	p.F31L	Exon1		Missense	TW: 0.008	4. ‐	4. VUS	AR
							gnomAD: 0.0007			
							1000G: 0.0014			
							TW: 0.005506			
							gnomAD: 0.0003			
							TW: 0.0045			
33	TG	c.1348delT	p.S450	Exon9	Heterozygous	FS	gnomAD: 0.0000558	1. ‐	1. Pathogenic	AR
	TG	c.7404G>C	p.K2468N	Exon42	Heterozygous	Missense	TW: 0.001	2. ‐	2. VUS	AR
							gnomAD: 0.00000398			
35	DUOX2	c.3693+1G>T	‐	Exon28	Heterozygous	Splicing	gnomAD: 0.0002	1. VUS	1. Pathogenic	AR
	DUOX2	c.3967G>A	p.A1323T	Exon30	Heterozygous	Missense	1000G: 0.0006	2. VUS	2. VUS	AR
							TW: 0.0015			
							1000G: 0.0002			
							TW: 0.001003			
37	DUOX2	c.2635G>A	p.E879K	Exon20	Heterozygous	Missense	gnomAD: 0.0000756	1. Pathogenic	1. Pathogenic	AD AR
	DUOX2	c.1772T>C	p.F591S	Exon15	Heterozygous	Missense	TW: 0.001	2. VUS	2. VUS	AD AR
	DUOX2	c.959T>C	p.L320P	Exon9	Heterozygous	Missense	TW: 0.0005	3. VUS	3. VUS	AD AR
							1000G: 0.0002			
							TW: 0.0005			
38	DUOX2	c.2654G>T	p.R885L	Exon20	Heterozygous	Missense	gnomAD: 0.0005	1. Conflicting	1. VUS	AD AR
	DUOX2	c.2048G>T	p.R683L	Exon17	Heterozygous	Missense	1000G: 0.0008	2. Conflicting	2. VUS	AD AR
	DUOX2	c.4027C>T	p.L1343F	Exon30	Heterozygous	Missense	TW: 0.012	3. VUS	3. VUS	AD AR
							gnomAD: 0.0004			
							1000G: 0.0006			
							TW: 0.008			
							gnomAD: 0.0006			
							1000G: 0.001			
							TW: 0.014558			
40	DUOX2	c.4027C>T	p.L1343F	Exon30	Heterozygous	Missense	gnomAD: 0.0006	1. VUS	1. VUS	AD AR
	DUOX2	c.2654G>T	p.R885L	Exon20	Heterozygous	Missense	1000G: 0.001	2. Conflicting	2. VUS	AD AR
	DUOX2	c.2048G>T	p.R683L	Exon17	Heterozygous	Missense	TW: 0.014558	3. Conflicting	3. VUS	AD AR
							gnomAD: 0.0005			
							1000G: 0.0008			
							TW: 0.012			
							gnomAD: 0.0004			
							1000G: 0.0006			
							TW: 0.008			
41	TG	c.1348delT	p.S450	Exon9	Heterozygous	FS	gnomAD: 0.0000558	1. ‐	1. Pathogenic	AR
	TG	c.4628G>C	p.C1543S	Exon22	Heterozygous	Missense	TW: 0.001	2. ‐	2. VUS	AR
							gnomAD: 0.00000398			
42	TG	c.274+2T>G	‐	Exon3	Heterozygous Heterozygous	Splicing	TW: 0.0005	1. ‐	1. VUS	AR
	TG	c.1348delT	p.S450	Exon9		FS	gnomAD: 0.0000558	2. ‐	2. Pathogenic	AR
							TW: 0.001			
43	TPO	c.670_672del	p.D224del	Exon7	Heterozygous	Inframe deletion	TW: 0.0005	1. ‐	1. VUS	AR
	TPO	c.2268dupT	p.E757*	Exon13	Heterozygous	Nonsense	gnomAD: 0.0001	2. Pathogenic	2. Pathogenic	AR
44	TPO	c.2268dupT	p.E757*	Exon13	Homozygous	Nonsense	gnomAD: 0.0001	Pathogenic	Pathogenic	AR
45	DUOX2	c.3329G>A	p.R1110Q	Exon25	Heterozygous Heterozygous	Missense	gnomAD: 0.0002	1. Pathogenic	1. VUS	AD AR
	DUOX2	c.2635G>A	p.E879K	Exon20		Missense	1000G: 0.0004	2. Pathogenic	2. VUS	AD AR
							TW: 0.0025			
							gnomAD: 0.0000756			
							TW: 0.001			
46	TSHR	c.1135C>T	p.Q379*	Exon10	Heterozygous	Nonsense	‐	1. ‐	1. ‐	AR
	TSHR	c.1349G>A	p.R450H	Exon10	Heterozygous	Missense	gnomAD: 0.000235	2. Conflicting	2. VUS	AR
							1000G: 0.0002			
							TW: 0.002503			
50	DUOX2	c.2048G>T	p.R683H	Exon 17	Heterozygous	Missense	gnomAD: 0.00462107	1. VUS	1. VUS	AR
	DUOX2	c.4027C>T	p.L1343F	Exon 30	Heterozygous	Missense	TW: 0.008	2. Conflicting	2. VUS	AR
	DUOX2	c.3709_3711dup	p.S1237dup	Exon 29	Heterozygous	Inframe insertion	gnomAD: 0.00750245	3. VUS	3. VUS	AR
							TW: 0.0145			
							gnomAD: 0.00152224			
							TW: 0.002			

Abbreviations: ACMG, American College of Medical Genetics and Genomics; AD, autosomal dominant; AR, autosomal recessive; CADD, combined annotation dependent depletion; DANN, deleterious annotation of genetic variants using neural networks; gnomAD, genome aggregation database; MAF, minor allele frequency; MetaLR, meta logistic regression; MetaSVM, meta support vector machine; Polyphen2_HVAR,D, polymorphism phenotyping v2; HumVar training set; SIFT, sorting intolerant from tolerant; TW, Taiwan biobank; VEST3, Variant Effect Scoring Tool version 3; VUS, variant of uncertain significance; 1000G, 1000 Genomes Project.

Additionally, comparison of term and preterm patients with the identified gene variants showed no statistically significant difference in thyroid function between the two groups (Table 5).

**TABLE 5:** Subgroup analysis comparing term and preterm patients positive for genetic variants.

	Term, positive for genetic variants (mean, standard deviation)	Preterm, positive for genetic variants (mean, standard deviation)	*p*
TSH (mIU/L)	107.8, 108.3	212.4, 198.9	0.090
T4 (μg/dL)	6.0, 2.8	3.6, 1.4	0.084
T3 (ng/dL)	126.3, 41.0	84.5, 44.3	0.059

## 4 DISCUSSION

Previous studies on TDH found predominantly *TG* gene mutations in Western populations, while *DUOX2* mutations were more common in Asian countries.^13^ Consistent with these findings, in our Southern Taiwanese cohort, *DUOX2* mutations were the most prevalent, followed by *TG*, *TSHR*, and *TPO* mutations. However, the recurrent hotspots identified in our study differed somewhat from those identified in populations that immigrated from Mainland China. We identified several recurrent mutations that were mostly found in East‐Asian populations, including *DUOX2* c.3329G>A, R1110Q (13.3%), *TSHR* c.1349G>A, R450H (11.1%), *TG* c.1348delT, S450 (8.9%), and *TPO* c.2268dupT, E757* (8.9%). This finding contrasts with that found in Malaysian Chinese populations, where the c.2268dupT, E757* mutation in the *TPO* gene was the most common mutation (33%) in TDH cases,^14^ compared to 8.9% in our cohort. Additionally, the *DUOX2* R1110Q mutation accounted for only 2.2% in mainland China,^15^ while in our cohort, it accounted for 13.3%. These results suggest that within Asian Chinese populations, there are regional differences in the genetic variations associated with TDH that might be related to demographic shifts. In our study, we observed a high percentage of TDH (40%) in permanent CH cases, which is consistent with several Asian studies that show an increasing incidence of TDH.^5^, ^6^ This finding might result from the increased number of preterm infants in a teaching hospital setting. We identified a total of four novel variants among the three apparent CH patients, as indicated by significantly elevated TSH levels at initial diagnosis. Two of the four novel variants are located in the *TSHR* gene. *TSHR* c.1135C>T is a nonsense mutation predicted to result in premature protein truncation resulting in a classic loss‐of‐function mutation, while *TSHR* c.1349G>C is a missense mutation causing an arginine to proline change at the conserved residue 540. With this mutational hotspot, a change in cysteine to histidine is pathogenic. The other two novel variants were found in the *TG* gene. *TG* c.2459T>A is a missense mutation that causes an amino residue change from phenylalanine to tyrosine at position 820. *TG* c.2461delA is predicted to be pathogenic in multiple in silico analyses. This frameshift mutation generates a stop codon at position 821, causing truncated TG protein before the functional cholinesterase‐like domain that is required for folding and secretion of thyroglobulin.^16^, ^17^ These two variants are presented in cis and located in proximity, raising the possibility that they are evolutionarily co‐transmitted. However, further investigations including population genetic analyses are needed to confirm this hypothesis.

The significant difference in birth body weight between the positive and negative gene variant groups may be attributed to the higher proportion of preterm births in the negative gene variant groups, consequently leading to a lower birth body weight. Our study also observed a lower rate of genetic variant detection in preterm TDH patients compared to term TDH patients (50% vs. 70.6%) (Table 6). The incidence of CH in preterm infants is reported to be higher than in term infants,^18^ as factors other than genetic mutations interfere with thyroid hormone synthesis. These factors include underdevelopment of the thyroid gland, immaturity of the hypothalamic–pituitary–thyroid axis, as well as environmental and medical interventions. In our study, we encountered an extremely low birth weight infant who was born at 24 weeks of gestational age and suffered from permanent CH without any identified variant genes. This patient was initially treated as having Transient Hypothyroxinemia of Prematurity based on a normal TSH level and low free thyroxine level after birth. She was later diagnosed with CH at 4 months of age under the impression of delayed TSH elevation during a routine screening in the neonatal intensive care unit and started levothyroxine treatment. However, at 5 months old, the patient underwent surgery for necrotizing enterocolitis, which led to the development of short bowel syndrome (SBS). In this case, the evolution into permanent CH may have been associated with SBS, resulting in dietary restrictions and subsequent iodine deficiency.^19^ Furthermore, although our subgroup analysis showed no statistically significant differences in TSH and thyroxine levels between term and preterm infants with identified genetic mutations, a trend was observed in which preterm infants had lower T3 and T4 levels and higher TSH values. This trend may be related to the relatively insufficient thyroid hormone reserves commonly seen in preterm infants.^18^ The trend warrants further confirmation in a larger population base in future studies. In conclusion, these findings emphasize the complexity of thyroid hormone metabolism in preterm infants.

**TABLE 6:** Presence of genetic variants for TDH in preterm vs term patients.

Presence of genetic variant	Term (*N* = 34)	Preterm (*N* = 6)
Positive	24 (70.6%)	3 (50%)
Negative	10 (29.4%)	3 (50%)

The treatment of CH is not contingent on identifying the genetic etiology. However, a previous study reported that genetic testing influenced the treatment decisions for patients with permanent CH, allowing the cessation of thyroxine therapy in cases of TDH or thyroid hypoplasia.^20^ Upon reviewing our TDH patients, those without pathogenic variants attempted to discontinue levothyroxine therapy, but were unsuccessful. For example, patients 1, 25, 32, 47, and 49 unsuccessfully attempted to discontinue levothyroxine therapy twice before the age of six. Patients 3, 12, 19, and 38 demonstrated poor compliance with their prescribed thyroxine regimen, leading to elevated TSH levels. Patients 32 and 36 each made a single attempt to discontinue thyroxine before turning six. Patient 15, after discontinuing thyroxine therapy at the age of three, experienced a significant rise in TSH levels to 177.6 mIU/L, preventing further attempts at medication cessation. These unsuccessful attempts to discontinue medication in these TDH patients suggest the involvement of unidentified genes in thyroid hormone synthesis. Furthermore, several patients in our cohort harbor heterozygous mutations in the *TSHR*, *TPO*, and *TG* genes, including P5 and P6, P14, P30, and P44. The technical limitations of high‐throughput sequencing could explain the absence of a second mutation in these patients. Although autosomal recessive in inheritance, heterozygous loss‐of‐function mutations in genes such as *TSHR* and *TPO* are reported to result in a milder phenotype.^14^, ^21^, ^22^ Therefore, further attempts to discontinue medication may be considered for the four patients in the future.

This study has several limitations that should be considered. First, our patient cohort was predominantly composed of individuals from Southern Taiwan, potentially reducing the applicability of our findings to the entire Taiwanese population. This geographic concentration may affect the generalizability of our results. Second, the relatively small sample size of our study may have limited the statistical analysis power, preventing the detection of finer genetic variations in the population, and possibly leading to an overestimation of the effects of certain genetic variants. However, as our hospital operates as a single‐center referral medical facility specializing in the treatment of CH, serving an approximate population of 3 million, the results still hold a certain degree of representativeness.

Our study excluded TDH patients receiving follow‐up care in the adult endocrinology department. This exclusion criterion may have led to selection bias, potentially affecting the comprehensiveness of our findings. Lastly, the lack of parental sequencing data in our study limits investigation into the potential effects of digenic mutations.

In conclusion, a WES analysis of 53 TDH‐related genes in 45 Taiwanese TDH patients revealed a mutational spectrum in the Southern Taiwanese population, with *DUOX2* mutations being the most common. Patients with causative variants identified through WES accounted for 71.1% of all TDH cases, which is higher than previously reported, highlighting its utility in identifying genetic causes of TDH. As more evidence emerges, it is anticipated that our understanding of the genetics underlying TDH will increase, potentially leading to modifications in the approach to lifelong treatment.

## CONFLICT OF INTEREST STATEMENT

All authors declare no conflict of interest.
